# Bibliographical Notices

**Published:** 1846-03

**Authors:** 


### BIBLIOGRAPHICAL NOTICES.

*Medical and Physiological Problems, being chiefly Researches for correct Principles of Treatment in disputed points of Medical Practice.* By WILLIAM GRIFFIN, M. D., Member of the Royal College of Surgeons in Edinburgh, one of the Physicians to the County of Limerick Infirmary, Consulting Physician to the Lying-in Hospital, &c.; and by DANIEL GRIFFIN, M. D., Member of the Royal College of Surgeons in London, Assistant Physician to the County of Limerick Infirmary, one of the Physicians to the Lying-in Hospital, &c. 8vo. pp. 359. London, 1845.

We rejoice to meet occasionally with a production from the press, which contains the results of observation and reflection on important topics appertaining to medicine. Yet we are rarely favoured. Since the "Notes" of Dr. Holland were published, we have seen none such. They were most favourably received by the thinking portion of the profession—alas! how few in number—yet they did not answer, in this country, the purposes of the publishers to the extent they ought to have done; and hence we have no new issue reprinted from the second London edition. This last we have the good fortune to possess, and know how to value it. The mass of purchasers look for what they consider the most *practical* works,—forgetting, that no correct practice can exist without a due scientific appreciation of every department of medicine. Were it otherwise, as well might we listen to the results of the empiric, who can *affirm* as strongly as, and more strongly than, the physician the success caused by the administration of his nostrums. *Principia non homines* was a motto—of very questionable latinity—adopted by one who wished to be regarded as the adopter of principles and not of men. It was wisely suggested, that *principia et homines* was more judicious; and so would we say of "facts not principles"—a motto absurdly chosen by a class of modern observers as an index of their rule of action. Let it be "*facts and principles;*" or rather "*principles and facts;*" for assuredly the establishment of great principles to guide us in the management of disease is of infinitely greater importance than searching after isolated facts,—unless such search be undertaken by those who are eminently capable. It is proverbially difficult to trace the relations between cause and effect. It requires a mind well trained in psychical exercises; and yet we greedily devour, and implicitly credit, statements in the journals on evidence totally insufficient to convince a jury, or, indeed, a single individual whose mind has been properly disciplined in the search after truth. Let a formula for the cure of hemorrhoids be published, and instantly it is copied into every journal at home and abroad; whilst a careful and praiseworthy inquiry into the value of a medicinal agent, which leads to its being shorn of any reputed virtue, and to the beneficial results ascribed to it being transferred to great principles of hygiene, passes by unheeded.

The work before us consists of "principles *and* facts;" and the very first paper is a good specimen of the whole. It is entitled "*Inflammation of the Bowels treated by Opium.*" We hail its appearance with pleasure, because it confirms the view, which we have always embraced, that opium, in *proper doses*, is one of our most valuable agents in inflammatory affections. When given in small quantities, it is an undoubted excitant. The error has been to regard it as such when administered in large doses. It is then unquestionably a sedative, and one of the best that we possess. In painful inflammatory and other affections, its influence is most precious. Not only does it positively diminish the activity of the organic actions; but it blunts the sensibility of the nervous system, and prevents the irritation that would necessarily arise from nervous irradiations in painful disorders, which tend so much to aggravate or complicate the original malady.

In inflammation of the bowels its use has been thought to be contraindicated, under the belief, that it has a constipating effect, and may therefore augment the mischief in sero-enteritis; yet such is by no means the fact. By reducing the inflammation, the action of the bowels will be restored; and if opium be capable of accomplishing this, then its effects are rather laxative than constipating. In endo-enteritis, however, or inflammation of the mucous coat, this objection to opium—if well founded—could not apply, for in it there is increased secretion from the lining membrane. Principles and facts have proved to us, after a long experience, that it is equally suited for both these pathological conditions. It is, we repeat, in appropriate doses, a precious sedative,—and therefore adapted for the removal of inflammation—mucous as well as serous. On this point we wish to speak emphatically; but then the remedy must be given freely. Our own course is to prescribe it in soft pill,—not less than two and a half or three grains; and if, in the course of four hours, the symptoms do not yield, to repeat the dose. Commonly we premise a full bleeding from the arm, and follow this immediately by the opium. The value of the combination is now almost universally admitted; and let the observation of Dr. Armstrong—acknowledged to have been a most painstaking and excellent practitioner—be borne in mind—that if in enteritis he were told, that he must rest his hopes of safety on the lancet or on opium, he would unhesitatingly prefer the latter. "So great," says he, "is my confidence in full doses of opium in peritoneal enteritis, that if compelled to say, and supposing myself the subject of the disorder, whether I would exclusively rely upon them solely, or upon bloodletting solely, I should certainly fix upon the former; at the same time I should like to have the simultaneous influence of both remedies, being convinced they are by far more serviceable combinedly, than separately employed."

It has been already remarked, that opium, by removing the enteric inflammation, may restore the alvine evacuations, which have been withheld owing to such inflammation. It has been a question, however, whether cathartics should not be given also. It need scarcely be said, that where parts are inflamed, anything that is calculated to irritate them should be carefully withheld. No one would doubt the truth of this as a general principle, nor can we readily imagine the special case that should form an exception. Yet by the routinist, cathartics have been advised to meet a mere epiphænomenon; for the constipation, in almost all cases of sero-enteritis, is the consequence rather than the cause of the phlegmasia.

It was a wise remark of Dr. William Saunders, that the best agent for opening the bowels in enteritis is the lancet; and upon the same principle we would urge opium, alone or in conjunction with the lancet; but it may admit of serious doubt, whether any active cathartic should ever be given. Dr. William Griffin, who is the author of Problem 1,—*What principles should be kept in view by the physician in the treatment of enteritis,*"—does not believe purgatives to be at all times unadvisable in enteritis.

"There are," he says, "occasions in which they are of use, even in the early stage; but it is difficult to offer indications by which we shall recognize these occasions in practice. In the advance of the disease, when its force is considerably broken, and the bowels may be supposed capable of acting without increasing or renewing the inflammation, there must be an obvious advantage in getting rid of the contents of the bowels, and this may perhaps be then generally effected with safety, by means of mild purgatives, combined with henbane. If, however, there was no injurious distension present, and the inflammation was progressively declining, my disposition would be to await a more perfect amendment before I would give even these."

And he subsequently adds:—

"General experience testifies, that the strongest purgatives will not operate in the early stages of inflamed bowels, unless large depletion by the lancet has been premised,—that is, unless the violence of the inflammation has been in some measure subdued: while on the other hand, as soon as this has been accomplished, they commonly occur spontaneously, or with the assistance of the mildest purgatives.

"Notwithstanding the free operation of purgatives at an early stage of enteritis, the inflammation may proceed to a fatal termination unless arrested by other remedies.

"A purgative has been known to occasion inflammation of the bowels, and when inflammation has been subdued by other remedies, it has brought on a recurrence of it.

"Inflammation of the bowels may be perfectly subdued without any evacuation at all.

"The bowels may even sometimes continue in a confined state for three or four days after the inflammation has subsided, without occasioning injurious distension." p. 34.

All this is discriminating; and experience of some length leads us to declare, that it perfectly accords with "principles and facts!"

The next problem, by the same gentlemen, is—*How are nervous affections distinguishable from inflammatory?*" Dr.

William Griffin has been long known for his views on spinal irritation, of the existence of which nothing either he or any one else has hitherto written, has carried conviction to our mind. Nor is the present paper more convincing. His view on this important topic of differential diagnosis is the following:

"In all acute inflammations of vital organs, I believe that no spinal tenderness will be found, except where it existed previous to the supervention of the attack," [he adds in a note, "some writers on spinal irritation state that they have found spinal tenderness with inflammation of liver,"] "or where the spinal cord itself happens to be the seat of such inflammation. In all neuralgic affections, on the contrary, tenderness of some portion of the spinal column, *usually that corresponding to the affected organ*, may be detected, except in some rare cases in which it seems probable the ganglionic nerves alone are concerned. As these cases must still present a difficulty in their diagnosis, we must rest contented with those general characteristics, which, however vague, or liable to lead us into error, are all we have to guide us, and all we have hitherto had to determine our opinion in that large class of neuralgic affections, for the detection of which I have been offering a new, and I believe, less doubtful sign."

Spinal irritation is a hobby with the author; and one that he has ridden somewhat unmercifully, with spur and whip. We are not surprised, consequently, at his attaching undue importance to it. No one, not similarly prepossessed, could have ventured to lay down so inadequate a test in doubtful cases; and we question much, whether Dr. Griffin himself is not rather guided in his judgment by more rational concomitant phenomena. In this case we want from him both "principles and facts."

Problem third, by the same author,—"*what is the diagnosis of abdominal inflammations?*"—is an embodiment of the same notions that are comprised in Problem second; whilst Problem fourth gives his views at length on "*spinal irritation*"—on which we do not think it worth while to dwell—until the existence of the malady has been proved. As yet it may be esteemed wholly hypothetical.

The next problem—the fifth in the series—by Dr. D. Griffin, inquires "*under what circumstances, and to what extent, is bleeding proper in diseases of the brain?*"

The Author concludes by recapitulating the circumstances in which he considers large bleedings improper, with the principles on which, in his estimation, they are so.

" *First.* They are improper, because symptoms are no certain test of the amount of disease, and we may produce a degree of debility that cannot be contended with.

" *Secondly.* They are improper in all cases in which extensive disease of brain is known or suspected to exist, as in such cases, besides the immediate danger, they produce a degree of debility that would interfere with the process of reparation, so far as that is possible.

" *Thirdly.* They are improper in all cases of disease of brain attended with severe and protracted pain, as such cases usually die, not from any mechanical effect of an existing inflammation, but from the exhaustion produced by the pain that accompanies t.

" As a general rule, I would, therefore, even in circumstances in which the loss of a large quantity of blood was supposed to be necessary, prefer taking it away in moderate quantities at certain intervals, watching the progress of the case, and being guided entirely by it." p. 87.

Problem VI, by Dr. William Griffin, is " *On what morbid state does the occurrence of Coma and sudden death in Jaundice depend?*" In this we see nothing new. The author considers it to be superinduced by the " suppression of a most important excretion, as it sometimes is in the suppression of the catamenia, and almost always of the urine. When we find inflammation of the brain or its membranes suddenly brought on by the obstruction of the uterine or renal discharges, we cannot be surprised that a suppression of one of the most important in the whole system, whether as a secretion or an excretion, should occasionally be induced." p. 96.

In Problem VII, " *Is the law of visible direction, as at present received, a true one?*" Dr. D. Griffin objects—and we have long maintained the same opinion—that Sir David Brewster's views on the law of visible direction are not borne out by facts. It is now, indeed, passed over almost *sub silentio* by some of the best writers on optics.

An elaborate inquiry in Problem VIII, " *Is Laryngismus stri-*

*dulus, or the crowing disease, a spasmodic or paralytic affection?*" leads Dr. Wm. Griffin to the following summary of the amount of our present information, and of the facts connected with the disease.

"By the concurrent testimony of almost all who have noticed the affection, it occurs, for the most part, if not wholly, in strumous habits.

"It is frequently found in connexion with enlarged glands in the neck, and perhaps in the thorax.

"It is frequently found in connexion with eruptions on the face, ears or scalp.

"It frequently terminates in convulsions, and is sometimes, though very rarely, ushered in by them.

"It is met with in families in which children are subject to head affections or convulsions, but who have also the strumous disposition.

"It is sometimes met with in connexion with an apoplectic or comatose state from the commencement, as in the cases of crowing apoplexy, which I have described.

"In a great proportion of the cases which terminated fatally, there was no symptom of head affection through their whole course, beyond the occasional fits of breathlessness and crowing: and then the children were as well, apparently, a few moments before death, as they were previous to the first attack of the disease, or as any children could be.

"The complaint is sometimes, but rarely, attended by cough or permanent difficulty of respiration.

"I believe it may be said that from one-third to half of all the cases of which we have any account, terminated in death. But this great mortality may perhaps in some degree be attributable to the over-active treatment pursued in many instances, from mistaken notions of the congestive or cerebral nature of the disease." p. 140.

We pass over Problem IX, by Dr. W. Griffin, "*Does suffering necessarily imply self-consciousness? Are sentient beings necessarily percepient?*" which is an elaborate physiological inquiry,—and Problem X, by the same writer—"*What are the therapeutic effects of opium?*"—on which the author's views are sound, and well worthy of perusal; and proceed to Problem XI, also by Dr. W. Griffin, "*What principles should regulate the treatment of Hæmoptysis?*" One great object of the author is to deprecate the too constant employment of frequent depletion and protracted low regimen. He properly remarks, that in most cases hæmoptysis occurs in constitutions predisposed to tubercular phthisis, "and it is equally well known, *that in such constitutions tubercles are readily generated by any deterioration of the vital power.*" Another weighty objection, however, is, that repeated blood-letting communicates a tendency to the recurrence of the hemorrhage, especially if fluids be freely allowed. Loss of blood augments absorption; fluid readily passes into the vessels by imbibition; the blood is thus rendered thinner, and better adapted for escape by diapedesis.

In Problem XII, "*How should acute rheumatism be treated?*" Dr. W. Griffin discusses the opium and calomel treatment of Dr Hope, and the opium treatment of Dr. Corrigan. The latter prescribes opium in doses of one or two grains every second or third hour, or of ten or twelve grains or more in the twenty-four hours. It is not improbable, that the entire efficacy of Dr. Hope's plan is dependent upon the opium. "The most important rule to be remembered," says Dr. Corrigan, "in employing opium for the cure of acute rheumatism is, that *full and sufficient doses* shall be exhibited. I have heard of opiate treatment having disappointed those who have tried it. On inquiry, I have learned that in those cases it has been given only to the extent of a grain every fourth or sixth hour. This is not the treatment of rheumatism by opium; it is making the patient worse than before; it is inflicting on the patient the mischief arising from the stimulant effects of the drug, and withholding from him all the benefits of its sedative influence. The opium should always be increased in dose, both as to frequency and quantity, until the patient feels decided relief; and should be then kept up at that dose until the complaint is steadily declining."

The average time of cure under Dr. Corrigan's treatment is nine days. We can speak, from positive experience, of the marked value of opium in this affection. A case has just passed from our hands to a state of health, where not more than six grains were given in the course of the day; yet all the arthritic phenomena had disappeared by the end of the week.

The last paper is the joint production of the two Griffins. It is entitled "*Observations on the application of Mathematics to the Science of Medicine,*" in which the importance of medical statistics is properly urged,—but with no new views. To therapeutics it is confessedly difficult of application, and this very difficulty will prevent it from being had recourse to by the mass of the profession.

Such are the topics investigated in the work before us:—all of great interest; and some of deep moment. Excepting where the authors are carried away by preconceived notions—as in the case of spinal irritation—their views are generally sound, and manifestly the offspring of reflecting minds: and if they do not always add to our stock of knowledge, they are eminently suggestive, and may lead to farther investigation.

*General Therapeutics and Materia Medica, with one hundred and twenty illustrations. Adapted for a Medical Text Book.* By ROBLEY DUNGLISON, M. D., Professor of Institutes of Medicine, etc., in Jefferson Medical College of Philadelphia, formerly Professor of Materia Medica and Therapeutics, etc. etc. *Third edition, revised and improved. In two volumes,* 8vo. Lea & Blanchard: Philadelphia, 1846.

In these times of rampant empiricism and bold pretence, when so many commend themselves to favour by decrying all theory as idle speculation, and rejecting every thing but experience, and all experience but their own, it is encouraging to find a work devoted to the inculcation of principles in medicine so well sustained by the profession as the present. The short time which has elapsed since the publication of the former edition of this standard work, sufficiently testifies that there are still among us many who appreciate science, and are not yet disposed to worship the Baals of the day. Facts and principles must ever elucidate and sustain each other. No one is silly enough to contend for principles that are inconsistent or in direct opposition to well established facts—but facts must not be confounded with assumptions—nor the limited observations of individuals received as truths, which contradict the testimony of thousands who are equally competent to observe and impartial to declare. It is on this rock that so many split. In their eagerness to avoid unfounded hypothesis, they plunge into the blindest empiricism. In the appropriate language of our author:—

"A knowledge of the healthy and diseased functions, or of physiology and pathology, and of the ordinary effects of therapeutical agents on those functions, obtained by careful and repeated observation, must be the basis of that enlightened theory, which necessarily leads to enlightened practice; and great mischief would result to both, were we to discard all rational therapeutics, and restrict ourselves to mere observation. The complex functions, executed by the human organism, are so modified by multitudinous external and internal influences, which are inappreciable; so much agency is perpetually exerted by the *moral* over the *physique*, that no comparable facts can be obtained in sufficient number to admit of any accurate numerical deduction; and constantly, we must either treat disease in accordance with principles suggested by conjoined observation and reason; experiment for ourselves *ab initio;* or resign our faith to the asserted observation and experience of others; and of these, which of the legion shall we select as masters? It is fortunate that we are possessed of such principles in medicine. Without them we should be unable to meet morbid manifestations, which present themselves to us for the first time. 'He,'—says Dr. Abercrombie,—'who follows certain arts or practical rules, without a knowledge of the science on which they are founded, is the mere artizan or the empiric; he cannot advance beyond the practice-rules which are given him, or provide for new occurrences and unforeseen difficulties.

"These great principles are the same everywhere, and by their possession we can combat disease wherever we meet with it; amongst the equatorial heats, or the Siberian snows; in the scorching presidencies of British India, and *à fortiori* in every portion of this wide-spread territory; the lofty mountain, and the lowly valley; the pestiferous locality on the banks of the Mississippi, and the more salubrious regions where malarious influence is unknown. It is by their possession, that the medical officers of the army and navy know how to manage the diseases of all climes, when opportunity is offered them for adequate observation. That diseases are modified by climate or locality cannot be doubted, but the well instructed physician speedily seizes hold of the peculiarity."

The therapeutic principles advocated in this edition, are substantially the same as presented in the former, and constitute, in our estimation, the chief value of the work. We have had too many occasions for deploring the neglect, by otherwise well informed practitioners, of a proper attention to this subject, not to feel anxious that works of this character should be generally studied. Assuredly, nothing so much contributes to the multiplication and support of quackery, in its various phases, as the empirical practice of honorable members of the profession. By empirical practice we mean prescribing articles as specifics, or cures, for disease, whether one or many compose the prescription, without duly considering the pathology, and well understanding the physiological actions invoked or excited by the remedies employed—without, in short, a proper comprehension of what is to be expected of the natural offices of the system, and the kind and degree of control or assistance afforded by the treatment pursued. The pragmatical disposition to interfere on all occasions of disease,—to regard nature as always wrong,—that every thing is to be done by physic and nothing left to the recuperative powers of the system,—is the greatest fallacy prevailing among those of our art. Too little attention to hygiene and too much reliance upon perturbating remedies, has, we verily believe, done more towards weakening the confidence of the public in our noble profession, and strengthening the hands of Charlatans, than all the lying and trickery of these detestable knaves, favoured as they are by the weakness and credulity of their dupes.

The extensive circulation of the two former editions of this work of our author, and the manner in which its doctrines have been noticed by the leading journals of this country and Europe, renders it unnecessary for us to speak of the different parts of the work, or indeed of any part in detail. We cannot close these remarks, however, without expressing our great satisfaction with the highly practical views of the author on the subjects of bloodletting and the use of opium in the treatment of inflammation. The error of resorting to bloodletting in all inflammations, or of relying upon it exclusively, or nearly so, in any, as some do, is well set forth; as well as the unfounded prejudice of many against opium in conjunction with bloodletting, in inflammations involving important organs.

The Materia Medica portion of the work contains the usual amount and kind of information on that subject. All the principal articles and means of cure are treated of, briefly, but with sufficient detail for the purposes of either students or physicians. Scattered through the volumes, we have numerous wood-cut illustrations of medicinal plants, mostly well executed, which add to the value of the publication.

---

*The Natural History and Diseases of the Human Teeth.* By Joseph Fox, M. R. C. S. L., &c. First American from the third London Edition. Remodeled, with an introduction and numerous additions. By Chapin A. Harris, M. D., D. D. S., Professor of Practical Dentistry and Dental Pathology in the Baltimore College of Dental Surgery, etc. etc. Illustrated with Thirty Plates. Royal 8vo. pp. 440. Ed. Barrington & Geo. D. Haswell. Philadelphia. 1846.

Mr. Fox was originally a pupil of the celebrated surgeon, Mr. Cline, and served under him as a dresser in Guy's Hospital. Circumstances afterwards induced him to devote much of his time to the study of the teeth and their diseases; on which subjects he subsequently became a distinguished lecturer at Guy's Hospital. The first complete edition of his work, on the history and diseases of the human teeth, was published in 1806, and has continued to enjoy the highest reputation ever since. No work on the subject has ever appeared for which intelligent dentists have manifested a higher regard. The present edition comes forth with the advantages of a careful revision, and the sanction of a distinguished American teacher and practitioner of the dental art.

In preparing the work for republication in this country, the editor informs us that he has found it necessary to make numerous and extensive additions, in order to adapt it to the present state of dental practice. That these additions are judicious, we need no better guarantee than the known competency of the editor.

The mechanical execution of the present edition is in the highest degree creditable to the enterprising publishers.

